# Preliminary demonstration of benchtop NMR metabolic profiling of feline urine: chronic kidney disease as a case study

**DOI:** 10.1186/s13104-021-05888-y

**Published:** 2021-12-24

**Authors:** Natalie Finch, Benita Percival, Elena Hunter, Robin J. Blagg, Emily Blackwell, James Sagar, Zeeshan Ahmad, Ming-Wei Chang, John A. Hunt, Melissa L. Mather, Séverine Tasker, Luisa De Risio, Philippe B. Wilson

**Affiliations:** 1 University of Bristol Veterinary School, Langford House, Langford, Bristol, BS40 5DU UK; 2 Nottingham Trent University, Brackenhurst Lane, Southwell, NG25 0QF UK; 3 Oxford Instruments Magnetic Resonance, Tubney Woods, Abingdon, Oxfordshire OX13 5QX UK; 4 De Montfort University, The Gateway, Leicester, LE1 9BH UK; 5 Nanotechnology and Integrated Bioengineering Centre, University of Ulster, Jordanstown Campus, Newtownabbey, Northern Ireland UK; 6 University of Nottingham, University Park, Nottingham, NG7 2RD UK; 7 Linnaeus Veterinary Limited, Friars Gate, Solihull, B90 4BN UK

**Keywords:** Chronic kidney disease, Cat, Metabolite, Metabolomics, NMR

## Abstract

**Objective:**

The use of benchtop metabolic profiling technology based on nuclear magnetic resonance (NMR) was evaluated in a small cohort of cats with a view to applying this as a viable and rapid metabolic tool to support clinical decision making.

**Results:**

Urinary metabolites were analysed from four subjects consisting of two healthy controls and two chronic kidney disease (CKD) IRIS stage 2 cases. The study identified 15 metabolites in cats with CKD that were different from the controls. Among them were acetate, creatinine, citrate, taurine, glycine, serine and threonine. Benchtop NMR technology is capable of distinguishing between chronic kidney disease case and control samples in a pilot feline cohort based on metabolic profile. We offer perspectives on the further development of this pilot work and the potential of the technology, when combined with sample databases and computational intelligence techniques to offer a clinical decision support tool not only for cases of renal disease but other metabolic conditions in the future.

**Supplementary Information:**

The online version contains supplementary material available at 10.1186/s13104-021-05888-y.

## Introduction

Metabolic profiling involves the investigation of molecules within a biological system and the perturbations within that in response to internal or external stimuli. Metabolic profiling and associated techniques have been previously applied to human, animal, and plant models [1–7]. High-field nuclear magnetic resonance (NMR) and mass spectrometric measurements are considered the gold standard for metabolomic investigations [8–14], however, there are limitations with employing these techniques in point-of-care or near-patient settings, such as veterinary or medical practices, due to the mechanical and electrical requirements of such equipment. Benchtop NMR (bNMR) however provides an opportunity for the selectivity of NMR to be utilised and accessible to healthcare and veterinary professionals, providing a wealth of metabolic data to support clinical decision-making. bNMR-based metabolomics has recently shown potential to overcome human health challenges, for example, demonstrating use in point of care settings for human urinary analysis of type 2 diabetic (T2D) patients, with results acquired under 15 min [15–21]. Indeed, bNMR-based metabolic profiling can therefore be proposed as a translational healthcare technology to identify perturbations in metabolites from disease and the environment.

Typically, a multi-platform approach is used to diagnose conditions in felines inclusive of clinical symptoms and physical examination. At present, the diagnosis of felines for some elements of renal diseases are dependent on histology and are invasive procedures, requiring tissue biopsies [11]. However, cognitive biases are present upon observing tissues, and this is not a quantitative method of diagnosis due to the discipline predominantly being qualitative or semi-qualitative [11]. Alternative methods include the growth of aerobic bacteria using aerobic urine culture, which is time-consuming and has potential contamination risks [12]. Moreover, abdominal radiographs and ultrasounds can be used to support clinical examinations however these require owner intervention and physical intervention of the clinician at the surgery [12]. Furthermore, particularly over the COVID-19 pandemic period, veterinary consultations took place remotely either via teleconference or video link [19]. Diagnostic tests routinely performed are inherently invasive, and particularly complicated in a period of lockdown to contain a global pandemic, as well as being particularly difficult based on behavioural factors in some domesticated species; however, collecting material for urinary analysis is relatively facile and benchtop NMR, once clinically validated, offers the potential for highly rigorous remote investigations [21–25]. Practically, for such remote collections to be of value to the community, simple post-sampling steps can be implemented by owners; in a time where it is now commonplace to self-administer lateral flow tests, the post-collection stabilisation of the urine sample prior to transfer to a laboratory would only involve a sterilisation phase which could be simply carried out through addition of a pre-aliquoted solution (containing reagents described in *Experimental* section) to the collected urine sample. Therefore, to detect chronic kidney disease (CKD) in the early stages, changes in metabolites in urine (as a proximal sample) can be identified. Such analysis can provide a deeper understanding of the mechanisms and metabolic pathways key to the progression of CKD. Cats with CKD are staged according to guidelines developed by the International Renal Interest Society (IRIS) and accepted by the American and European Societies of Veterinary Nephrology and Urology. The IRIS stages range from no azotemia (IRIS stage 1) to the most severe azotemia (IRIS stage 4). Staging guidelines are helpful for making diagnostic, prognostic and therapeutic recommendations for CKD.

## Main text

Free-catch, fasted urine samples were collected from cats clinically diagnosed with CKD from urinalysis, GFR and serum biochemistry assessments and centrifuged immediately (3500 rpm at 4 °C for 15 min). Healthy subjects were recruited from the local institutional cat population and had no clinically diagnosed underlying health issues. All subjects were administered similar diets. The method of urine collection was identical to case subjects above. A minimum of 0.5 ml of urine is required for analysis. The supernatants were then stored at − 80 °C prior to analysis. NMR spectra were obtained on an Oxford Instruments X-Pulse 60 MHz benchtop NMR spectrometer, operating at + 40 °C. Samples were defrosted, and diluted by addition of 20% (by volume) deuterium oxide, D_2_O. One-dimensional proton NMR spectra (with and without solvent suppression), and proton-proton gradient-selective COSY spectra were obtained for each sample. One-dimensional ^1^H spectra were collected with 64 scans, 6 s acquisition time and 5 s relaxation delay; one-dimensional solvent suppressed ^1^H spectra [using a WET (Water suppression Enhanced through *T*_1_ effects) sequence] were collected with 128 scans, and the same acquisition time, and relaxation delay; COSY spectra were obtained with 8 scans of 256 slices. All spectra were internally referenced to H_2_O/HOD at δ_H_ + 4.66 ppm. The Kyoto Encyclopedia of Gene and Genomics (KEGG) was used to ascribe significant biomolecular modifications and describe linkages between metabolic cycles.

High-field metabolomics has already demonstrated translational capability for diagnostic and therapeutic aims in humans with renal conditions [26]. Herein, we use CKD as a case study for the application of this technology and present pilot data from four subjects consisting of two control (Subjects S2,S4) and two with CKD IRIS stage 2 (S1, S3). Subjects S2 and S4 were clinically assessed by a veterinarian for confounding conditions and assessment as control participants for this pilot study as healthy controls with no renal conditions and serum creatinine concentrations of < 145 μmol/L. Further details on participants availbale within data protection regulations is available in the Additional file 1.

Subjects S1 and S3 were diagnosed with azotaemic CKD at Stage 2 according to IRIS guidelines from their serum creatinine concentrations of 193 and 188 μmol/L, respectively. Additionally, these CKD subjects show stronger resonances in the aromatic region (signal 17) ascribed to hippurate and phenylacetylglycine aromatic protons. Increases in urinary creatinine have previously been confirmed in cats with CKD [5]. Creatine is essential for energy transfer to skeletal muscle through the formation of ATP. Renal dysfunction can lead to an increase in creatinine in urine; therefore, the level of creatinine in urine is a principal indicator of CKD.

**Fig. 1:**
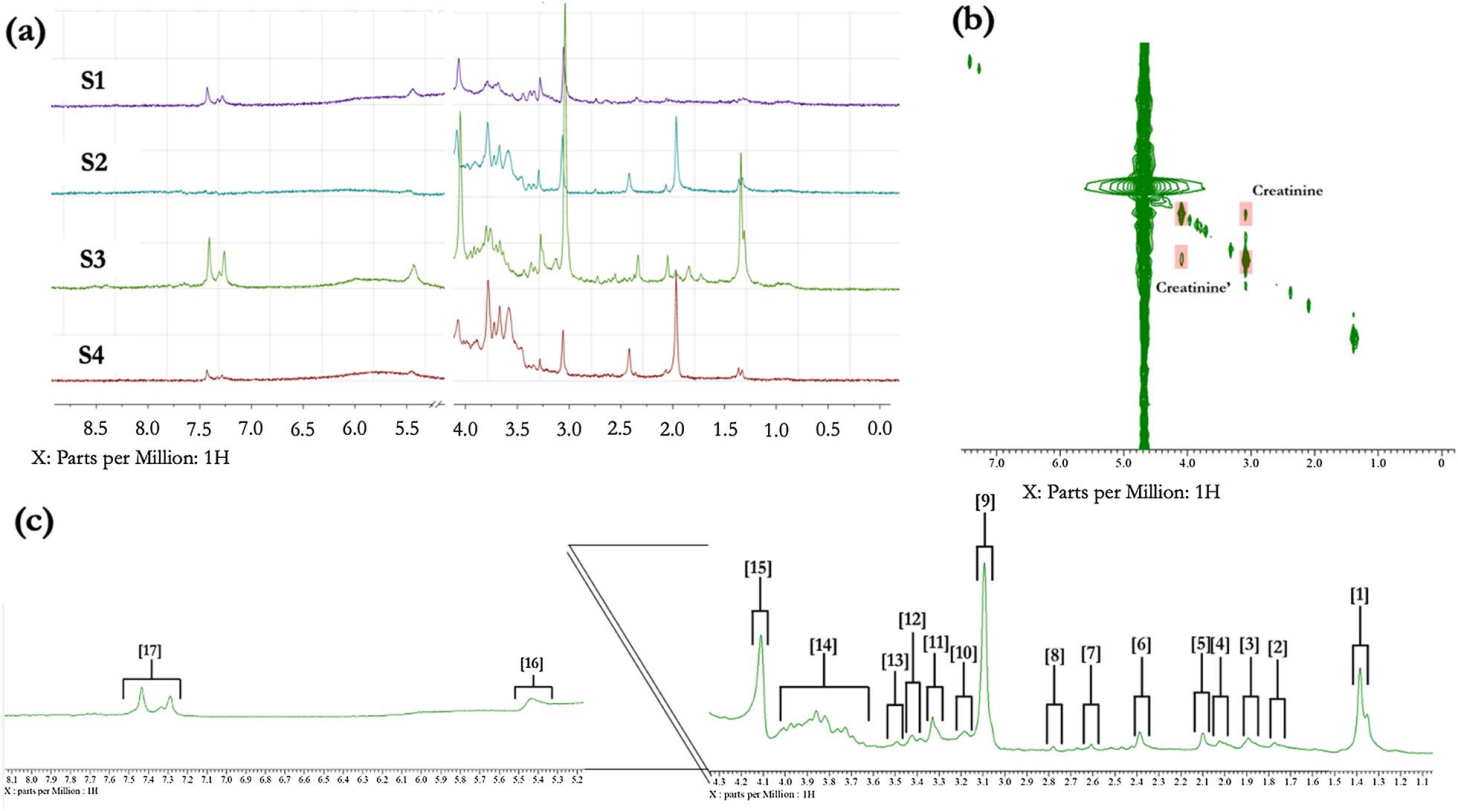
**a** urinary NMR metabolic profiles from feline subjects S1-S4 collected at 60 MHz operating frequency; **b** 2-dimensional COSY spectrum of signal confirmation for S3 sample showing creatinine cross-peaks; **c** assigned regions of S3 urinary profile with the following assignments: [1] 3-Hydroxybutyrate/Lactate-CH3/Felinine-CH3 [2] Tentative Felinine Derivative-CH3 [3] Tentative Felinine-CH2 [4] Acetate-CH3 [5] N-Acetyl [6] Pyruvate-CH3 [7] Citrate-CH2AB [8] Citrate-CH2AB [9] Creatinine/Creatine-N-CH2 [10] Felinine-CH2 [11] TMAO-N-CH3/Taurine-CH2/Betaine-CH3 [12] Taurine-CH2 [13] Glycine-CH2 [14] Felinine-CH2 [15] Creatinine-CH2 [16] Tentative Allantoin and Urea-NH2 [17] Aromatic signals consisting of Hippurate-CHs and phenylacetylglycine-CHs

Furthermore, the relatively weak acetate signal compared to healthy controls is indicative of decreased excretion in individuals with CKD, as displayed in Fig. 1 (S1, S3). Indeed, an inverse correlation between urinary excretion of acetate and renal function has been established in comparative physiological studies [4]. It has been observed elsewhere that the level of acetate was lower in humans with diabetes mellitus and CKD than those with CKD alone [13]. Reduced excretion of acetate in urine indicates further metabolism to acetyl coenzyme A which has a central role in fatty acid metabolism. Acetyl coenzyme A is involved in the central carbon metabolism that subsequently generates ATP through catabolism of the acetyl moiety in the tricarboxylic acid cycle [27]. Indeed, a reduction in urinary excretion of TCA cycle metabolites and renal expression of the genes which regulate these metabolites has been demonstrated in human cases of CKD, linking to mitochondrial dysfunction and CKD progression. Moreover, recent genomic and metabolomic assessments of human patients with non-diabetic CKD identified reduced TCA cycle activity in cases when compared to a control group. This reduction in urinary excretion of TCA cycle metabolites was linked to a reduction in overall mitochondrial biogenesis in kidney tissues from CKD patients likely caused by reduced expression of genes such as isocitrate dehydrogenase 3 in the tubointerstitial compartment of the kidney [9].

Metabolic pathway analysis (MPA) from these pilot data identified that glycine, serine and threonine metabolism was associated with classification between CKD and control subjects. An impact score from pathway topological analysis was 0.3, whilst a *p* value adjusted by the Holm-Bonferroni correction was 0.00426. This suggests that glycine, serine and threonine metabolism are modified in CKD subjects compared to the controls. Concentration decreases in metabolites excreted through both serine and threonine metabolism were detected elsewhere [10, 28]. Such a significance of serine metabolism between groups could be an indicator of its biological role in renal dysfunction. Serine acts as a mediator for methylation and the lowering of blood pressure in renal mechanisms. Serine excretion is correlated to glomerular filtration rate (GFR) which in-turn is used to define reductions in renal function. Therefore, if GFR is reduced, *D*-serine begins to accumulate in tissue [10, 15].

Furthermore, changes in glycine concentration in biofluids over the course of CKD has identified perturbations in amino acid metabolism in both rat and human models [18]. Additionally, hippuric acid metabolite signals in CKD subjects are linked to glycine conjugation with benzoic acid in hepatic, intestinal and renal activity [22]. Taken together, these glycine and glycine-conjugated metabolites are linked to oxidative stress and inflammation through both the IκBα/NF-κB and Keap1/Nrf2 pathways [23]. Moreover, glycine forms a central node in glutathione metabolism, which with tocopherol act as baseline markers of oxidation and concentrations of downstream metabolites are highly mediated by CKD stage. Cellular processes determining immunity, but particularly inflammation can be assessed by sphingolipid metabolites where sphingosines act as signalling molecules. These metabolites, also detected as N-acetyl functions (signal 5) have been demonstrated to be highly sensitive in their concentration to dietary interventions for the treatment of CKD in cats. Indeed, successful fibre supplementation aligned with positive clinical outcomes to changes in diet were ascribed to increased sphingolipid metabolite concentrations in plasma of cats with CKD [8].

Regarding other metabolites, the reduction of urine citrate concentration has also been associated with CKD where urine citrate can prevent the formation of calcium-based kidney stones [17]. Furthermore, the dysfunction of taurine was connected to CKD in other studies [2]. Taurine is involved in osmoregulation, calcium ion kinetics and regulation of the membrane potential in skeletal muscle. Moreover, taurine can be considered as anti-inflammatory and an antioxidant agent [25]. Kidneys have a crucial role in maintaining the level of taurine. However, the levels of taurine can be dramatically decreased in patients with CKD. Therefore, taurine can also feature one of the main regulatory metabolites for the detection of CKD [2].

Renal function can be assessed by the measurement of glomerular filtration rate and is often referred to as the gold standard, however this can involve clinically and technically challenging measurements. Indeed, CKD can be diagnosed in small animals through a combinatorial approach involving creatinine concentration and urine specific gravity, and whilst these may be widely used, they remain insensitive as prognostic and monitoring markers. Our preliminary assessment of a pilot feline cohort identifies (a) the ability of low field NMR spectroscopy to detect > 15 metabolites in feline urine and (b) the potential of the technology when applied to large cohorts and informed by machine learning, to provide fast biofluid analysis to support clinical decision making.

Benchtop NMR metabolic profiling offers an opportunity to leverage the selectivity of NMR spectroscopy in a portable format capable of being more widely applied, whilst taking advantage of chemometric methods to deconvolute spectra and offer the facility as a technique with diagnostic potential. Benchtop NMR is therefore a potential tool for the early detection of diseases and the evaluation of health conditions that can provide necessary, timely treatments [29, 30].

## Limitations

Whilst the authors are fully cognisant that the small sample sizes in this preliminary and pilot exemplar study do not allow for the substantiated development of a rationale for use in biomarker discovery or elucidation, we intend this article to act as a first case study or case report in the application of this miniaturised technology within the field of animal medicine. Indeed, further to our work in human medicine [20], a number of more specific feline metabolites (such as felinine) also need to be analytically quantified in terms of instrumental sensitivity in order to allow us to fully establish working thresholds for the technology in terms of limits of detection and quantification.

## Supplementary Information

**Additional file 1:** **Table S1.** Available data on study participants. **Table S2.** Labelled metabolites identified in urine from subjects S1–S4 and shown in manuscript Figure 1 (a)–(c).

## Data Availability

The datasets used and/or analysed during the current study available from the corresponding author on reasonable request.
