# Supplementary material for: Preliminary demonstration of benchtop NMR metabolic profiling of feline urine: chronic kidney disease as a case study
Source: BMC Res Notes. 2021 Dec 24;14:469. doi: 10.1186/s13104-021-05888-y (PMC8708514; doi:10.1186/s13104-021-05888-y)
Supplement: Supplementary file 1 — Additional file 1: Table S1. Available data on study participants. Table S2. Labelled metabolites identified in urine from subjects S1–S4 and shown in manuscript Figure 1 (a)–(c). [file 13104_2021_5888_MOESM1_ESM.docx]

SUPPLEMENTARY INFORMATION

**Preliminary Demonstration of Benchtop NMR Metabolic Profiling of Feline Urine: Chronic Kidney Disease as a Case Study**

**Table S1. Available data on study participants.**

| **Subject** | **Presentation** | **Case(IRIS stage)/Control** | **Serum creatinine concentration (μmol/L)** | **GFR** |
| --- | --- | --- | --- | --- |
| S1 | Male, neutered | Case (2) | 193 | Low |
| S2 | Male, neutered | Control | 145 | Normal |
| S3 | Female, neutered | Case (2) | 188 | Low |
| S4 | Female, neutered | Control | 141 | Normal |

**Table S2. Labelled metabolites identified in urine from subjects S1-S4 and shown in manuscript Figure 1 (a) – (c).**

| **Peak label** | **Assignment** |
| --- | --- |
| **1** | 3-Hydroxybutyrate/Lactate-CH3/Felinine-CH3 |
| **2** | Tentative Felinine Derivative-CH3 |
| **3** | Tentative Felinine-CH2 |
| **4** | Acetate-CH3 |
| **5** | N-Acetyl |
| **6** | Pyruvate-CH3 |
| **7** | Citrate-CH2AB |
| **8** | Citrate-CH2AB |
| **9** | Creatinine/Creatine-N-CH2 |
| **10** | Felinine-CH2 |
| **11** | TMAO-N-CH3/Taurine-CH2/Betaine-CH3 |
| **12** | Taurine-CH2 |
| **13** | Glycine-CH2 |
| **14** | Felinine-CH2 |
| **15** | Creatinine-CH2 |
| **16** | Tentative Allantoin and Urea-NH2 |
| **17** | Aromatic signals consisting of Hippurate-CHs and phenylacetylglycine-CHs. |
